# Exploring SARS-CoV-2 spike protein mutations through genetic algorithm-driven structural modeling

**DOI:** 10.1093/bioadv/vbaf288

**Published:** 2025-11-11

**Authors:** Valentina Di Salvatore, Avisa Maleki, Babak Mohajer, Alvaro Ras-Carmona, Giulia Russo, Pedro Antonio Reche, Francesco Pappalardo

**Affiliations:** Department of Drug and Health Sciences, University of Catania, Catania, 95125, Italy; Department of Drug and Health Sciences, University of Catania, Catania, 95125, Italy; Mimesis SRL, Catania, 95125, Italy; Department of Immunology, Faculty of Medicine, University Complutense of Madrid, Madrid, 28040, Spain; Department of Drug and Health Sciences, University of Catania, Catania, 95125, Italy; Department of Immunology, Faculty of Medicine, University Complutense of Madrid, Madrid, 28040, Spain; Department of Drug and Health Sciences, University of Catania, Catania, 95125, Italy

## Abstract

**Motivation:**

The rapid evolution of SARS-CoV-2 highlights the importance of computational approaches to explore mutational effects on the viral spike protein. In this work, we present a genetic algorithm (GA) framework applied to the structural optimization of spike protein variants, with a focus on energetic and binding properties rather than direct evolutionary prediction.

**Results:**

Our GA-driven pipeline generated spike variants with progressively improved structural stability as indicated by lower discrete optimized protein energy scores across generations. The approach also enabled evaluation of Gibbs free energy and binding affinity for spike—Angiotensin-converting enzyme 2 receptor interactions, revealing candidate conformations with favorable thermodynamic properties. These results demonstrate the algorithm’s capacity to refine protein models and explore mutational landscapes in silico, although no validation against naturally emerging variants was performed. This study presents a methodological framework for GA-based structural modeling of SARS-CoV-2 spike mutations. Rather than forecasting specific variants of concern, it demonstrates the feasibility of a computational approach that can be extended and integrated with evolutionary and experimental evidence to strengthen future efforts in variant monitoring and vaccine development.

**Availability and implementation:**

All the Python and R scripts are available upon request to the authors.

## 1 Introduction

RNA viruses, including SARS-CoV-2, mutate frequently due to the intrinsic properties of their genomes (Otto *et al.* 2021). The impact of these mutations on transmission and disease severity depends on mutation rates and their effects within, and between, hosts (Sanjuán and Domingo-Calap 2016, Sanjuán *et al.* 2010), contributing to the emergence of viral variants and influencing pandemic dynamics.

SARS-CoV-2 relies on its spike (S) glycoprotein, a homotrimeric class I fusion protein, for host cell entry. Cleavage by host proteases like furin and TMPRSS2 triggers conformational changes that enable Angiotensin-converting enzyme 2 receptor (ACE2) binding and membrane fusion (Ballesteros-Sanabria *et al.* 2022). Given this mechanism, accurately predicting variants of concern (VOCs) remains essential in infectious disease control.

Variants such as Alpha (B.1.1.7), Beta (B.1.351), Delta (B.1.617.2), and Omicron (B.1.1.529) emerged through spike mutations, increasing transmissibility, altering disease severity, and challenging vaccine efficacy. Meanwhile, mRNA vaccines like Pfizer-BioNTech and Moderna showed strong protection against the original strain, with booster doses later introduced to counter immune evasion by emerging variants (Ballesteros-Sanabria *et al.* 2022) (Harvey *et al.* 2021).

Forecasting new variants enables timely public health responses (Rashed *et al.* 2022), supported by early surveillance systems (Rashed *et al.* 2021). A comprehensive strategy integrating genomics, bioinformatics, phylogenetics, mutation tracking, epidemiology, and AI facilitates real-time analysis of viral evolution (Xu *et al.* 2022). In particular, understanding mutation-driven changes in spike–ACE2 binding and Gibbs free energy (Δ*G*) is key to anticipating variant behavior and biological impact (Bai *et al.* 2021).

Metaheuristic algorithms are widely used to solve complex problems in biology, economics, and engineering (Michalewicz and Schoenauer 1996, Holland 1992).

Among these, genetic algorithms (GAs) are a prominent population-based method inspired by natural evolution (Michalewicz and Schoenauer 1996). Proposed by J.H. Holland in 1992, it incorporates chromosome representation, fitness evaluation, and biologically inspired operators, including the inversion operator (Holland 1992). Chromosomes, often binary-encoded, are modified via crossover and mutation, while selection favors the fittest individuals. Through iterative application, GA drives the population toward optimal solutions (Katoch *et al.* 2021, Abou El Ela and Spea 2009).

One study proposed VOC-DL, a deep learning framework integrating variant data into models like VOC-LSTM, VOC-GRU, and VOC-BiLSTM to forecast daily COVID-19 cases. Using data from Italy, South Korea, Russia, Japan, and India over three months in 2021, all models performed well, with VOC-LSTM achieving the highest accuracy (average *R*^2^ = 96.83%). The authors concluded that VOC-DL, particularly VOC-LSTM, offers reliable long-term forecasting and could aid in assessing the impact of future variants (Liao *et al.* 2022). Another study predicted SARS-CoV-2 mutations using epidemiological, evolutionary, and immunological features. While effective at forecasting known mutations linked to ACE2 binding and immune escape, it could not anticipate novel mutations or full lineage evolution. Existing models, including machine learning and logistic regression, often fail to account for the structural effects of mutations on protein–ligand interactions, key factors in viral transmission and immune evasion (Bai *et al.* 2021).

In this study, we propose a GA-based computational framework designed to explore the structural and energetic implications of spike protein mutations. Rather than attempting to directly predict real-world variants of concern (VOCs), our goal is to demonstrate the feasibility of applying GA to optimize spike protein conformations and investigate their impact on stability and spike–ACE2 interactions. This methodological approach lays the groundwork for future extensions where genomic surveillance and evolutionary data could be integrated to assess predictive performance.

## 2 Methods

**Figure 1.**
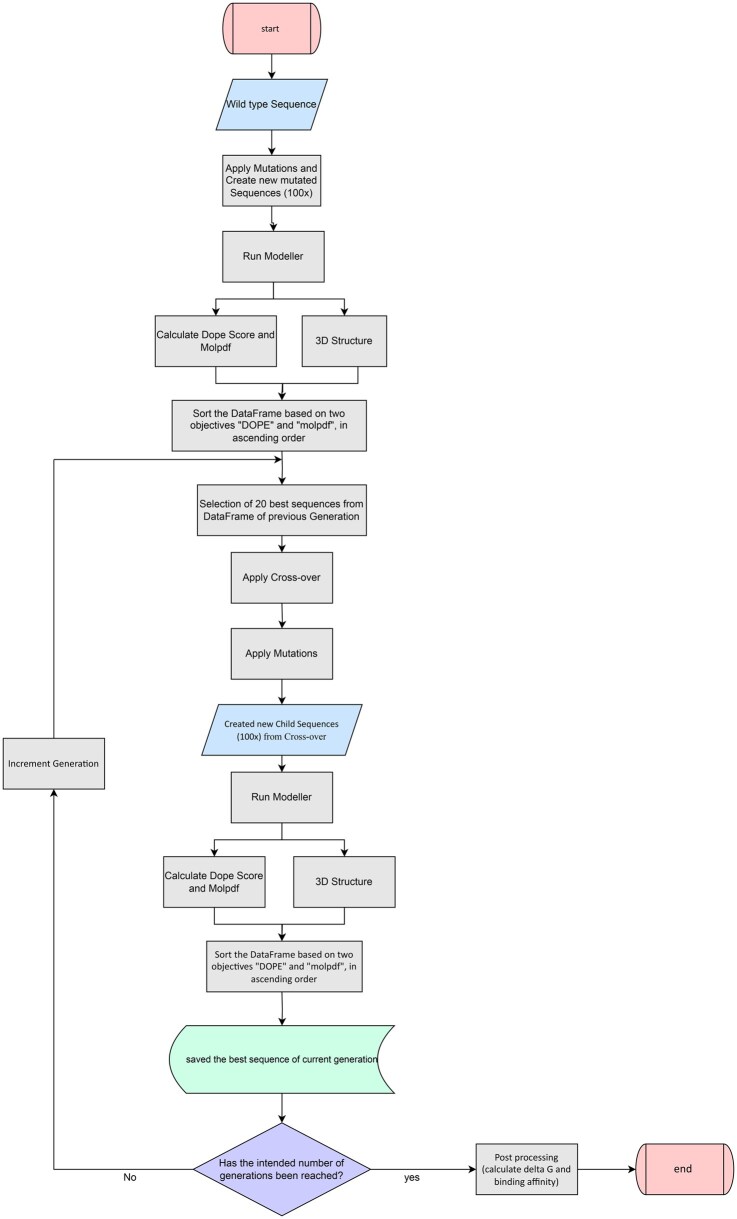
The workflow of the applied GA. A graphical representation detailing the step-by-step methodology used to predict new VOCs.

The prediction of VOCs is performed using GA optimization methods, which guide the search for optimal 3D structural models and sequences. This approach leverages the principles of natural selection and genetic evolution to optimize candidates for potential future VOCs. The workflow depicted in Fig. 1 illustrates the sequential steps involved in this predictive process:

- Population Initialization (Mutating Wuhan Spike Protein Sequences)
- Development of the Fitness Function (Energetic Metrics)
- Selection of the Best Fitness function (Select 20 top models)
- Cross-over (Pairwise Combinations of Top Models)
- Application of Mutations for Potential Better Models
- Gibbs Free Energy Calculation
- Binding Affinity Calculation

A statistical validation step was included to support the observed trend in discrete optimized protein energy (DOPE) score reduction across generations. Both parametric and non-parametric tests were employed to ensure the robustness and reliability of the results under different data distribution assumptions.

### 2.1 Mutating Wuhan spike protein sequences

We acquired the FASTA-formatted sequence of the Wuhan spike protein from the NCBI database (Accession ID: YP_009724390.1) and employed it as the input for an ad-hoc Python script implementing a GA. Using this code, we initially implement random mutations to 5% of the spike protein residues with a probability of 0.5. The 5% mutation rate is likely a compromise to introduce sufficient genetic variation without overwhelming the structure with too many changes, which could lead to biologically unrealistic models. This rate is intended to simulate a mutation level that could realistically be observed in viral evolution. A smaller mutation rate may not produce enough diversity to discover potential advantageous mutations, while a higher rate could destabilize the protein structure or create unrealistic conformations. Furthermore, this rate was selected as a methodological balance: higher values (>10%) risk introducing biologically implausible destabilization of the spike structure, while lower values (<2%) may not provide sufficient genetic diversity to explore alternative conformations. A 5% rate, therefore, ensures both computational feasibility and biological plausibility.

This iterative process was initially conducted for 100 generations, generating a unique sequence variant at each step. In addition, to further evaluate the robustness of the GA optimization, we extended the process to 1000 generations, as reported in the Results section.

### 2.2 Model building

Protein structure prediction was performed using MODELLER (https://salilab.org/modeller/), a widely used software for homology and comparative modeling of three-dimensional protein structures from amino acid sequences (Webb and Sali 2021). Based on the assumption that structurally similar proteins share similar sequences, MODELLER uses known structures of homologous proteins as templates to infer the target protein’s structure (Webb and Sali 2016). The modeling process includes fold assignment, sequence-template alignment, 3D model building, and structure evaluation. To assess model quality, we used MODELLER’s built-in scoring functions (Webb and Sali 2016, Šali and Blundell 1993):

- DOPE Score (discrete optimized protein energy) is a statistical potential used in homology modelling to assess protein structure quality. Implemented in MODELLER, it evaluates the energy of predicted models based on a reference state of non-interacting atoms in a spherical space. DOPE provides both overall and residue-level energy profiles, helping identify low-quality regions in the model (Eramian *et al.* 2006).
- MolPDF Score is an energy-based scoring function used to evaluate and refine protein structures. It assesses how well a predicted model fits experimental data, such as electron density maps from X-ray crystallography. Lower MolPDF values indicate better agreement between the model and experimental observations, suggesting a more accurate and reliable structure (Shen and Sali 2006).

These scores guided the selection of the most reliable structural models.

MODELLER was employed to generate structural models for 100 mutated sequences, obtained in the previous step, using the human ACE2 sequence retrieved from the RCSB Protein Data Bank (RCSB PDB). For homology modelling, the spike-ACE2 complex structure (PDB ID: 6M0J) was selected as the template. MODELLER aligned each mutated sequence to the template and predicted the corresponding three-dimensional structures. The resulting models were further refined through energy minimization algorithms to enhance structural stability and accuracy.

### 2.3 Selection of the best fitness functions

In genetic algorithms, selection directs the search toward optimal solutions. This study adopts Roulette wheel selection, which assigns selection probability based on fitness, maintaining diversity better than tournament selection and offering faster convergence than rank-based Selection, particularly useful for variable populations like SARS-CoV-2 spike variants.

From 100 modeled spike protein structures, the 20 with the lowest combined DOPE and MolPdf scores were selected via a weighted-sum multi-objective approach.

Both metrics were normalized according to the same formula:


$$O_{i,\mathrm{norm}}=\frac{O_{i}-O_{i,\min}}{O_{i,\max}-O_{i,\min}},$$

where *i* = 1, 2 denotes the two metrics (O_1_ = DOPE, O_2_ = MolPDF).

The weighted fitness function was then computed as:


$$O_{\mathrm{weighted}}=w_{1}\;O_{1,\mathrm{norm}\;}+w_{2}\;O_{2,\mathrm{norm}}$$

with *w*_1_ = 1 and *w*_2_ = –1. In this configuration, DOPE serves as the primary structural criterion, while subtracting MolPDF helps stabilize the optimization trend and reduce noise.

The choice of retaining the top 20 candidates reflects a balance between diversity and computational tractability. A smaller set would risk premature convergence toward suboptimal solutions, while a larger set would significantly increase computational costs. Twenty candidates ensured sufficient diversity for crossover operations while keeping the simulations computationally efficient.

### 2.4 Top models cross-over

Crossover was applied to the 20 top-ranking sequences selected via weighted multi-objective optimization, balancing genetic diversity and computational efficiency. A total of 100 offspring were generated through 50 crossover events, using repeated pairings among the 20 parents. This ensured broad parental contribution and promoted variability. Each offspring inherited traits from two parents, potentially combining beneficial features. This strategy enhanced exploration of the solution space and increased the likelihood of improved DOPE, MolPDF, and structural properties in the resulting models.

### 2.5 Mutation

Following the cross-over operation, diversity was instigated through a mutation process. Each residue had a 0.001 probability of being altered. The 0.001 probability for altering individual residues during the mutation phase is designed to ensure mutations remain conservative, minimizing the risk of detrimental mutations that could significantly impact structural integrity. This probability was chosen to maintain conservative evolutionary pressure. Larger probabilities would likely accelerate destabilizing changes, while smaller ones would reduce exploration efficiency. A mutation rate of 0.001 per residue thus represents a trade-off between the stability and the need for gradual exploration of the fitness landscape.

The resulting 100 new sequences underwent modelling, following the procedure outlined in the modelling section, to generate, and save, the best sequence of the current generation. This operation was repeated for 100 generations in the main experiment, with the best sequence from each generation saved as a representative. An extended 1000-generation run was also performed to assess long-term convergence and confirm the stability of the optimization trends.

### 2.6 Gibbs free energy evaluation

We modeled spike–ACE2 complexes for each mutant and estimated Δ*G* changes in protein–ligand binding using a surface area-based computational method. For each residue, surface atoms were identified based on a reference percentage of solvent-accessible area, capturing the regions most exposed to the environment.

We then calculated the change in accessible surface area (ΔASA) for each residue, reflecting surface differences before and after binding. These ΔASA values were used to estimate binding free energy (Δ*G*) via the following linear model (Schön *et al.* 2011):


$${\Delta G}_{\mathrm{bind}}=C+\;w_{\mathrm{Tyr}}{\;\Delta \mathrm{ASA}\;}_{\mathrm{Tyr}}+\;w_{\mathrm{Ser}}{\;\Delta \mathrm{ASA}\;}_{\mathrm{Ser}}+\;w_{\mathrm{Cys}}{\;\Delta \mathrm{ASA}\;}_{\mathrm{Cys}}$$

Here, C and the weights ($w_{\mathrm{Tyr}}$, $w_{\mathrm{Ser}}$, and $w_{\mathrm{Cys}}$) are predefined constants for TYR, SER, and CYS residues, which contribute most significantly to Δ*G*.

### 2.7 Binding affinity evaluation

Another important measure in the context of protein–ligand binding interactions in our work is the binding affinity. It is a measure of the strength of the interaction between a protein and a ligand and is quantified by the association constant (Ka). Ka represents the equilibrium between the bounded and unbounded states of a protein–ligand pairs:


$$K_{a}=\frac{\left[P][L\right]}{\left[PL\right]}$$

A higher Ka means stronger binding affinity. This implies that at equilibrium, more of the protein–ligand complex ([PL]) is formed relative to the concentrations of free protein ([*P*]) and free ligand ([*L*]). Thus, a higher Ka indicates that the binding reaction is more favorable and most of the protein and ligand molecules are in the bound state rather than unbound.

To describe the Ka mathematically, of binding affinity using the gas constant (R), and the temperature (T) in Kelvin (Kastritis and Bonvin 2013):


$$K_{a}=\exp\left(\frac{-\Delta G}{RT}\right)$$

Both the values of Δ*G* and binding affinity provide us with complementary information for understanding the protein–ligand interactions. Additionally, the agreement between Δ*G* and binding affinity values serves us in this study to validate the accuracy of the computational methods applied.

### 2.8 Select variants of concern

To identify potential VOCs, we applied a multi-criteria selection strategy based on three complementary metrics: DOPE score, Δ*G*, and binding affinity. The DOPE score was used as the primary fitness function within the genetic algorithm, enabling the selection of structurally stable and energetically favorable spike protein models. After generation, each model was further evaluated for its binding potential to the ACE2 receptor by calculating Δ*G* and binding affinity values. This combined assessment allowed us to select the most promising variants, those that not only exhibit low energy conformations (suggesting structural realism) but also demonstrate strong and thermodynamically favorable interactions with the host receptor. Based on this integrated analysis, specific generations were selected and introduced as candidate VOCs for further investigation.

### 2.9 Statistical analysis

To statistically validate improvements during GA optimization, we performed two analyses. First, we compared DOPE scores from the first and last 10 generations using both unpaired t-tests and Mann–Whitney U tests to account for potential non-normality and unequal variances, common in evolutionary algorithm outputs (Nachar 2008). Second, we applied the same tests to predicted Δ*G* and Ka values to evaluate enhancements in spike–ACE2 binding affinity and stability. The consistent results from both parametric and non-parametric tests confirmed the reliability of observed trends.

Although MolPDF is available in MODELLER, it was excluded due to its lack of normalization and dependence on modeling parameters, limiting its comparability. In contrast, DOPE is length-independent, more widely used in structural benchmarking (Šali and Blundell 1993), and better suited for evaluating diverse models. For these reasons, DOPE was chosen as the sole structural metric for validation.

All statistical analyses were conducted using the SciPy library (SciPy 1.0 Contributors *et al.* 2020), which offers reliable tools for hypothesis testing in Python.

## 3 Results

We used our GA workflow to explore the structural impact of mutations in emerging VOCs. Starting from the Wuhan reference spike protein, we introduced random mutations and evaluated their effects using a custom GA framework. The DOPE score served as the fitness function, guiding optimization toward energetically favorable models. Structural models were generated with MODELLER, and selection favored variants with minimized DOPE scores.

In total, 100 generations were initially produced, with each generation retaining the model exhibiting the lowest DOPE score. To further evaluate the structural refinement achieved through the GA, both DOPE and MolPDF scores were tracked across generations and compared to baseline values. This allowed for a comprehensive assessment of model improvement throughout the evolutionary process.

**Figure 2.**
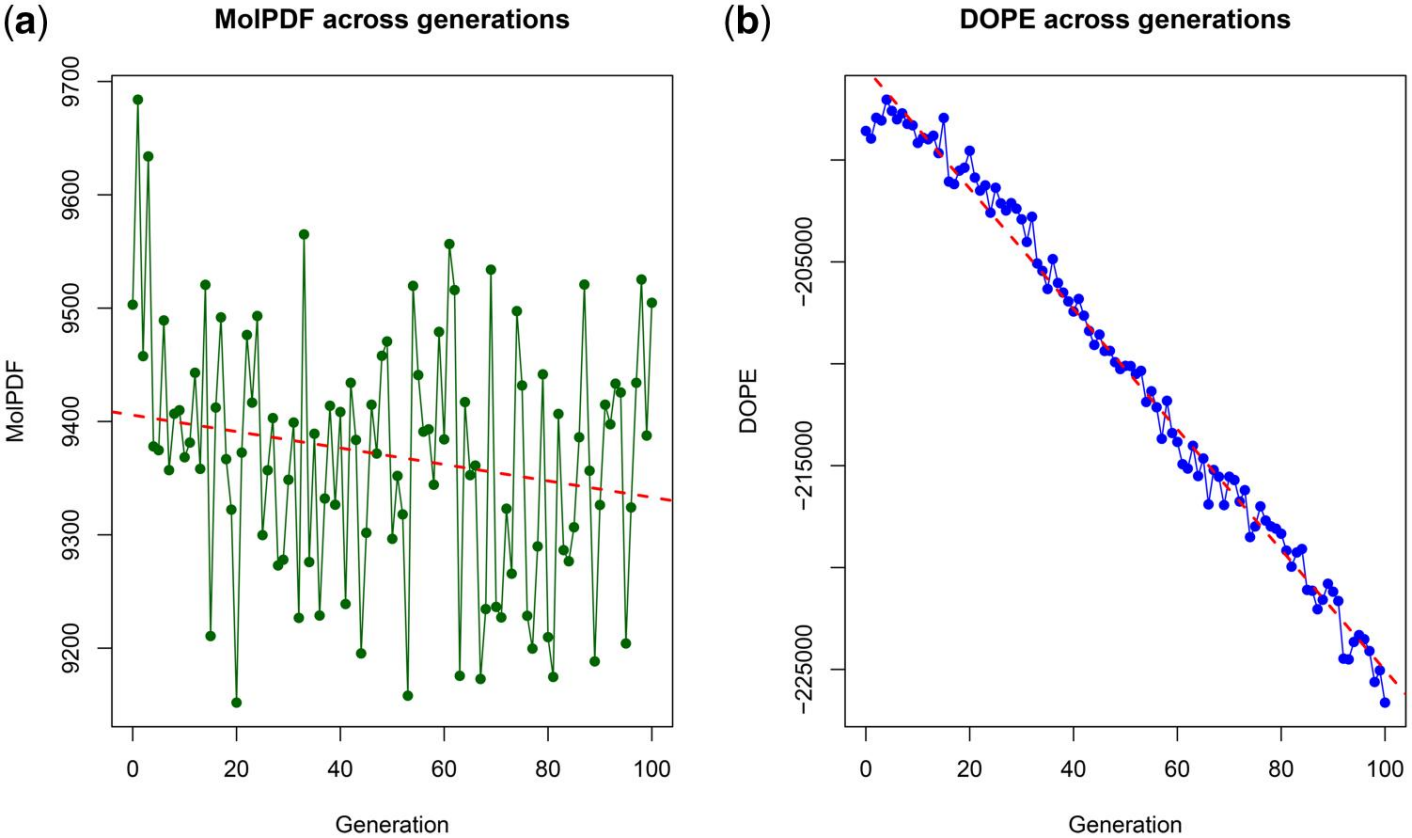
(a) shows the MolPDF score trend (green line with points). Although values remain highly variable across generations, the red dashed regression line indicates a slight overall downward tendency, suggesting limited but consistent structural refinement. (b) shows the DOPE score trend (blue line with points), which exhibits a clear and steady decrease across generations. The red dashed regression line highlights the strong convergence toward progressively more stable and energetically favorable structures. Together, these results demonstrate the effectiveness of the GA-driven optimization in refining spike protein models.

As shown in Fig. 2, DOPE scores steadily decrease across 100 generations, indicating improved model quality and structural stability. Since lower DOPE values correspond to more energetically favorable structures, this trend confirms the GA’s effectiveness in selecting better variants. MolPDF values remained highly variable across generations, with fluctuations that make the trend less apparent than for DOPE. Nevertheless, a slight overall downward tendency can be observed, suggesting limited but consistent structural refinement.

Together, these reductions highlight the robustness of the GA-driven optimization process in refining spike protein models through iterative selection.

**Figure 3.**
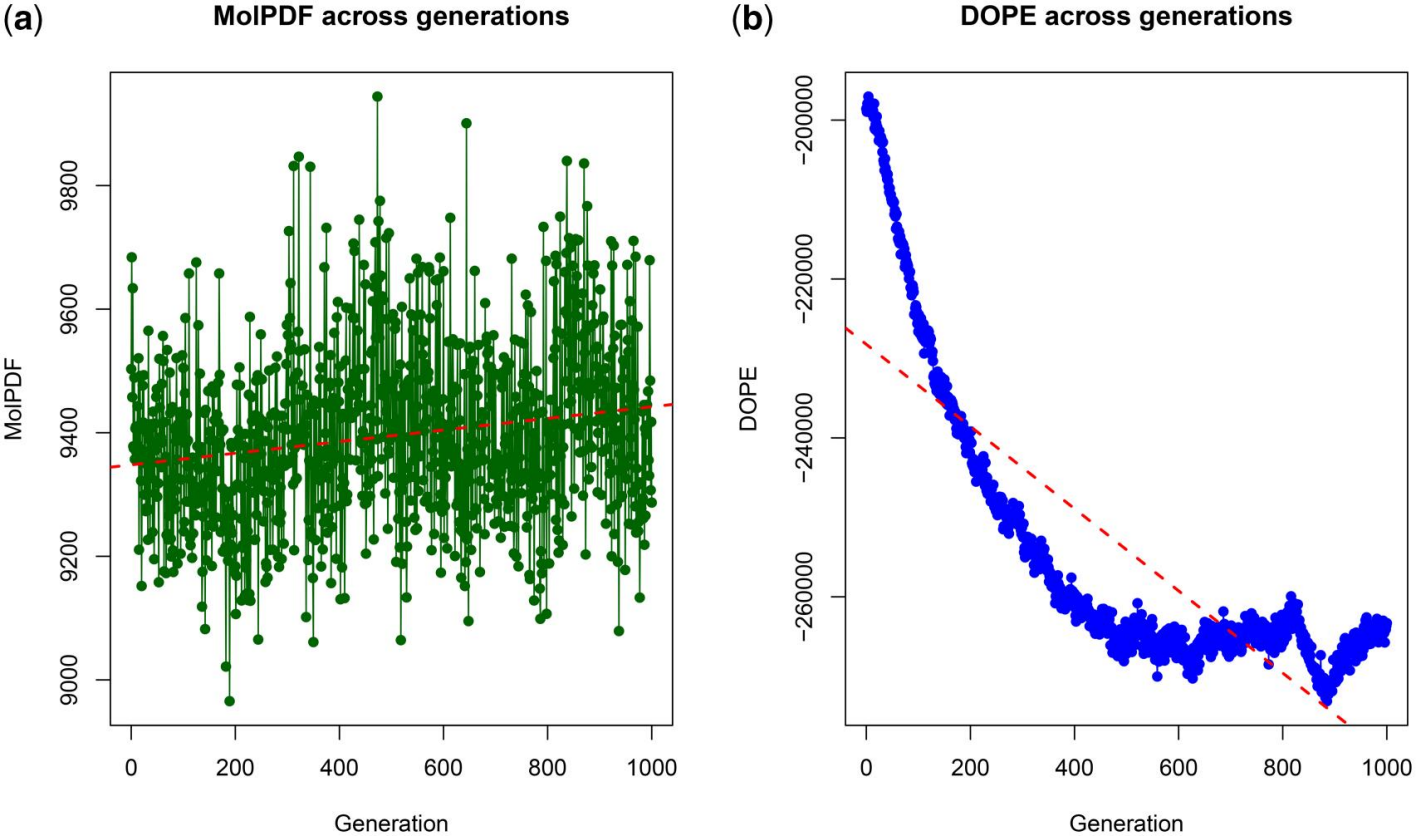
MolPDF (a) and DOPE (b) score trends across 1000 generations. The MolPDF values (green line with points) remain highly variable, though the red dashed regression line indicates a subtle overall decrease, consistent with limited structural refinement. DOPE values (blue line with points), instead, display a marked downward trend. Notably, after an initial steady decline, the DOPE curve shows a temporary plateau beyond ∼400 generations before resuming its descent, suggesting phases of stabilization followed by renewed optimization toward energetically favorable conformations.

To further validate the robustness of the GA, we extended the evolutionary process to 1000 generations (Fig. 3).

Consistent with the results from the 100-generation experiment, DOPE scores showed a strong overall decrease, reflecting the convergence of the GA toward progressively more stable and energetically favorable spike protein models. Interestingly, after ∼400 generations, the DOPE curve exhibited a temporary stabilization phase before resuming its decline, suggesting that the GA may encounter local optima during the search, from which subsequent generations allow escape and further refinement. In contrast, MolPDF values, which had shown a slight decrease in the 100-generation run, displayed higher variability and even a subtle upward trend across 1000 generations. This apparent discrepancy reflects the different sensitivity of the two metrics: MolPDF, as an internal pseudo-energy function of MODELLER, captures improvements during the early refinement phase but becomes less correlated with global structural stability over extended evolutionary runs. DOPE, instead, steadily decreases across both experiments, confirming its role as the most reliable indicator of GA-driven optimization.

Together, the results from both the 100- and 1000-generation experiments demonstrate that the GA framework consistently drives optimization toward lower-energy structural states, thereby confirming its suitability for modeling the impact of spike protein mutations. These structural improvements were then complemented by the thermodynamic evaluation of spike–ACE2 interactions (Δ*G* and Ka), providing a comprehensive assessment of both energetic stability and binding affinity across GA-driven evolutionary trajectories.

The Δ*G* values determine whether a process or reaction will occur spontaneously, while the Ka is a parameter used to study the binding affinity. Binding affinity typically involves assessing the strength of the interaction between a molecule (ligand) and its target (receptor), indicating how strongly they bind to each other.

**Figure 4.**
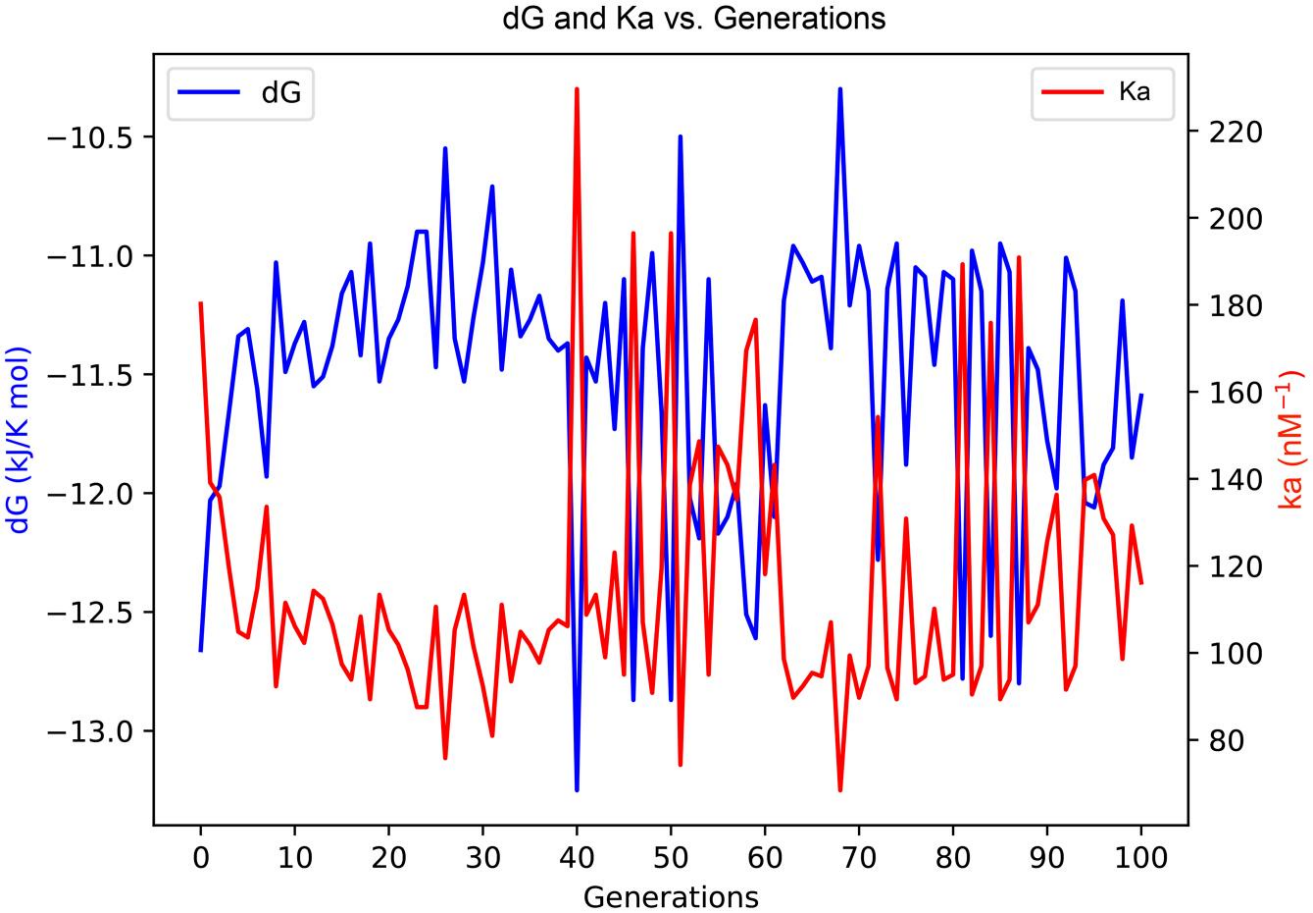
The plot illustrates the thermodynamic optimization of the spike–ACE2 interaction across generations. The Δ*G* values (blue curve) fluctuate but overall show a trend toward lower free energy, indicating improved binding spontaneity. The Ka values (red curve) also fluctuate, with occasional increases suggesting enhanced binding affinity. These results demonstrate the genetic algorithm’s capability to explore the fitness landscape and improve both the thermodynamic stability and functional interaction of spike protein variants.

Figure 4 illustrates the thermodynamic evolution of the spike-ACE2 interaction over 100 GA-optimized generations. While both Δ*G* and Ka fluctuate, the overall trend suggests a progressive shift toward more favorable binding conditions. Δ*G* values range from approximately −10.5 to −13.0 kJ/mol, with a general tendency toward more negative values, indicating increasingly spontaneous and thermodynamically favorable interactions. Concurrently, Ka exhibits substantial variability without a consistent trend, though intermittent peaks suggest episodes of enhanced binding affinity. These fluctuations reflect the GA’s capacity to explore diverse variants and occasionally achieve improved spike–ACE2 binding properties.

The trends in Δ*G* and Ka confirm that the GA enhanced not only structural quality (via DOPE and Molpdf) but also the thermodynamic and functional properties of spike–ACE2 binding. Lower Δ*G* indicates more favorable binding, while higher Ka reflects stronger affinity. These complementary improvements validate the robustness of our GA-based framework and highlight generation 97 as a promising candidate for a potential new variant of concern.

**Figure 5.**
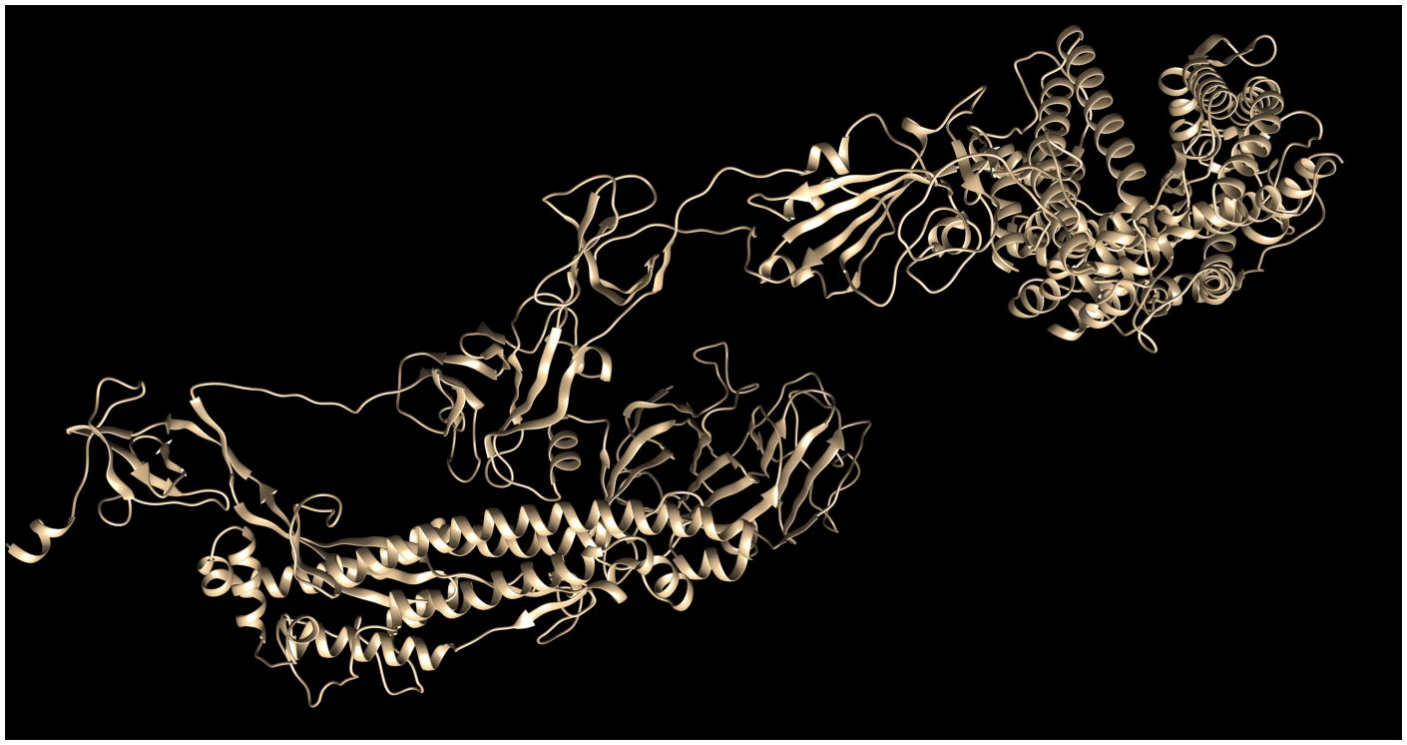
3D structure of spike protein of generation 97 obtained from the GA framework.

Generation 97 emerged as the most optimized candidate within our GA framework, displaying the lowest DOPE score and therefore the highest structural stability among the variants generated (Fig. 5). It also showed favorable Δ*G* and binding affinity values (Table 1), supporting its structural plausibility and efficient receptor engagement in silico. While these findings highlight the ability of the GA to identify structurally optimized spike variants, no claim is made regarding their occurrence in circulating lineages or their classification as real variants of concern.

**Table 1.** Performance metrics of the optimized model at Generation 97.

Generation	DOPE score	Δ*G*	Binding Affinity
97	−223520.296	−12.61	176.552

The table shows the key performance metrics for selected possible new VOCs. The columns include the generation number, DOPE score, Δ*G*, and binding affinity.

### 3.1 Statistical analysis

**Figure 6.**
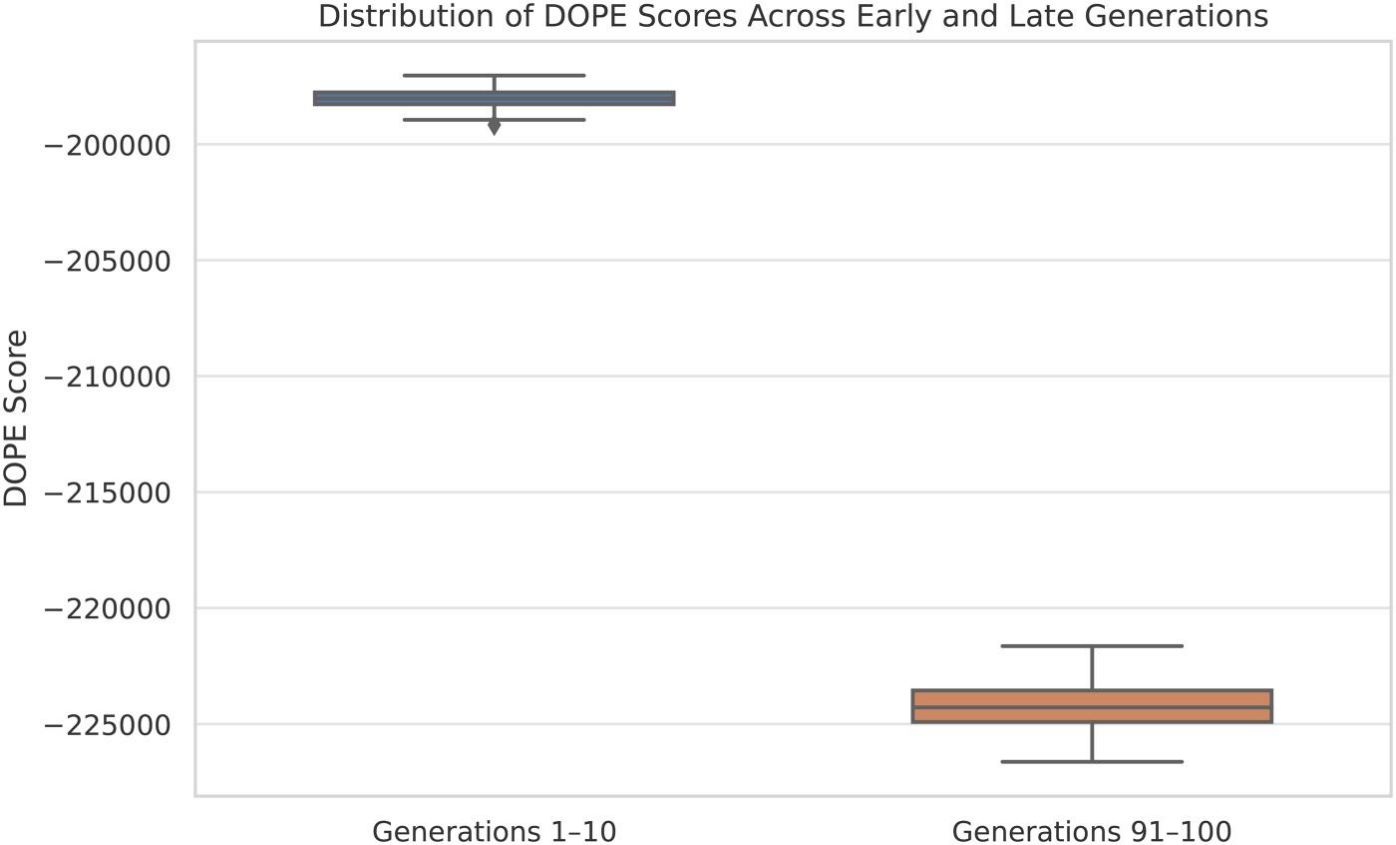
Boxplot of DOPE scores for the first and last ten generations. The significant decrease in DOPE values in later generations reflects improved structural stability driven by the genetic algorithm optimization.

A comprehensive statistical comparison was performed between the first and last ten generations of spike protein models to assess improvements in structural and functional metrics. As shown in Table 2, the DOPE score exhibited a highly significant decrease (*t*-test *P* = 2.36 × 10^−16^; Mann–Whitney *P* = .000183), confirming a progressive enhancement in model stability and conformational quality across generations. The median DOPE score decreased by more than 26,000 units between the first (−198,031.51) and last (−224,283.43) ten generations, reflecting a substantial gain in structural stability achieved through GA-driven optimization. Figure 6 illustrates this difference, showing the marked reduction in DOPE scores between early and late generations. This trend aligns with the core objective of the GA, which was designed to iteratively select variants with lower energy profiles, thereby promoting convergence towards structurally optimized models.

**Table 2.** Statistical comparison of spike protein models.

Test	*t*-test *P*-value	Mann-Whitney *P*-value
DOPE Score	2.36E−16	.00018267
Δ*G* (Gibbs Free Energy)	.20046886	.24114463
Ka (Binding Affinity)	.1992096	.24114463

The table shows *a* statistical comparison of DOPE score, Gibbs free energy (Δ*G*), and association constant (Ka) between early (generations 1–10) and late (generations 91–100) spike protein models.

**Figure 7.**
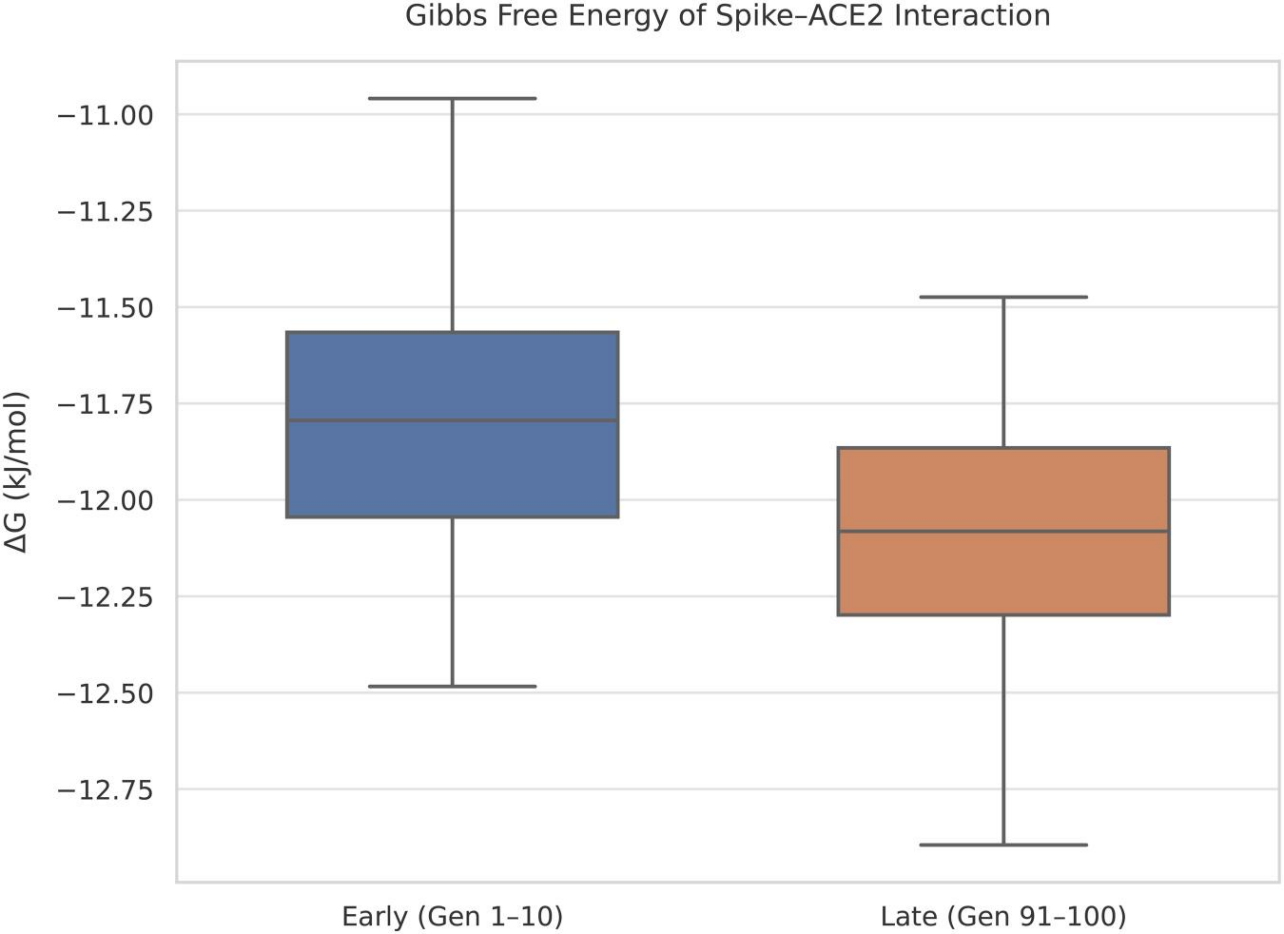
Δ*G* values of spike–ACE2 interaction across early and late generations. No statistically significant changes were observed, suggesting limited functional evolution under structural optimization pressure.

**Figure 8.**
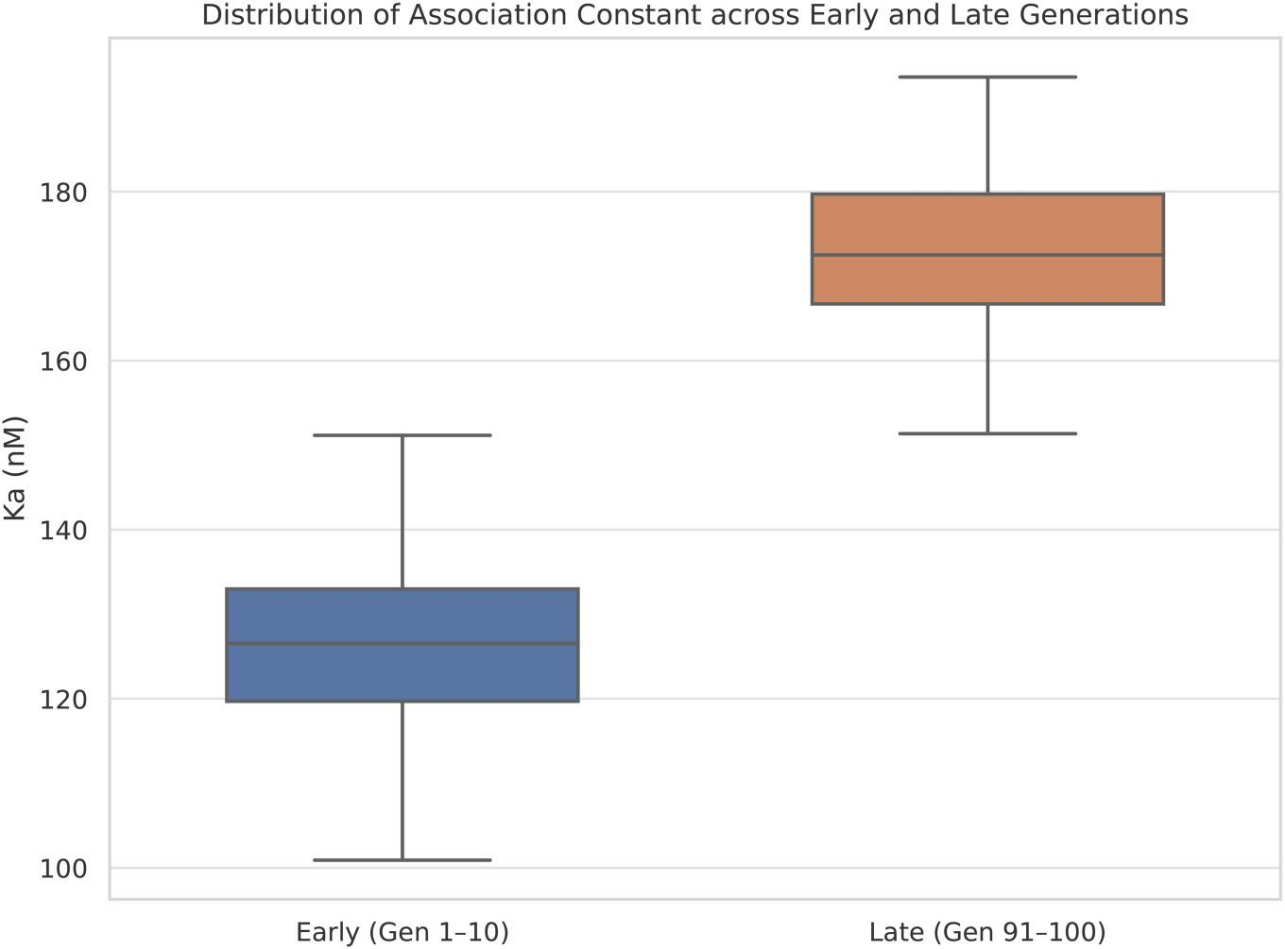
Ka distribution across early and late generations. The lack of significant variation indicates that binding affinity was not effectively optimized in the absence of direct selective pressure.

In contrast, the Δ*G* and Ka did not show statistically significant differences between the first and last generations (Δ*G P* = .20; Ka *P* = .20, *t*-test), as illustrated in Figure 7 and Figure 8. These results suggest that, while the GA effectively optimized structural stability, the functional improvements in binding thermodynamics were less pronounced. This interpretation is further supported by the modest differences observed in the mean values: Δ*G* improved only slightly, from −11.73 to −12.05 kJ/mol, and Ka increased from 125.50 to 143.40 nM^−1^ between the early and late generations. These limited shifts reinforce the notion that, while structural energy minimization was prioritized and successfully achieved, thermodynamic binding properties did not undergo a comparable evolutionary pressure.

A possible explanation lies in the multi-objective nature of protein–ligand interaction optimization. Δ*G* and Ka are influenced by subtle, non-linear conformational and surface changes that may not evolve in parallel with structural compactness or energy minimization (Van Den Noort *et al.* 2021). Moreover, as Δ*G* and Ka were not directly included in the GA’s fitness function but evaluated a posteriori, their evolution was not subject to selective pressure. This decoupling may explain the lack of statistically significant improvement in these functional indicators, despite the evident refinement of the structural model itself.

Together, these statistical results validate the effectiveness of the GA-driven modeling and selection strategy in optimizing structural features of spike protein variants. While functional metrics such as Δ*G* and Ka did not show significant improvements, the marked enhancement in structural stability supports the utility of this approach for generating conformationally optimized candidates with potential biological relevance.

## 4 Discussion

The use of genetic algorithms (GAs) guided by fitness metrics such as the DOPE score provides a flexible and powerful computational framework for exploring the mutational landscape of the SARS-CoV-2 spike protein. Post hoc analyses of Gibbs free energy and binding affinity offer complementary insight into structural stability and functional relevance, particularly in the context of spike–ACE2 interactions. While Δ*G* informs on thermodynamic feasibility, binding affinity reflects the strength of receptor engagement. Together with structural optimization, these measures help characterize the potential impact of mutations at the molecular level.

In our study, the DOPE score was employed as the main fitness function, and we consistently observed a progressive decrease across generations, suggesting convergence toward structurally more stable and energetically favorable models. Within the initial 100 generations explored, generation 97 exhibited the lowest DOPE score and represented a point of convergence in the optimization process. Extending the evolutionary process to 1000 generations further confirmed this trend, with DOPE scores continuing to decline and showing strong convergence overall. Interestingly, after approximately 400 generations, the DOPE curve entered a temporary plateau before resuming its descent, suggesting that the GA may encounter local optima during the search but is ultimately able to escape and refine further. This extended experiment reinforces the robustness of the GA-driven optimization framework and highlights its potential for long-term convergence.

MolPDF values, in contrast, displayed more variable behavior. While a slight decrease was observed in the first 100 generations, the extended 1000-generation run revealed higher variability and even a modest upward trend. This apparent discrepancy can be explained by the different sensitivity of the two metrics: MolPDF, as an internal pseudo-energy function of MODELLER, captures improvements during the early refinement phase but becomes less correlated with global structural stability over extended evolutionary timescales. Thus, while MolPDF remains informative, DOPE provides a more reliable and consistent measure of convergence and model quality in our GA framework.

Although Δ*G* and binding affinity did not exhibit statistically significant changes across early and late generations, they provided valuable additional context. The observation that structural stability improved more consistently than binding properties suggests that different selective pressures may govern these features and highlights the importance of multi-objective optimization in future applications. Importantly, combining binding-related parameters with structural stability metrics ensures that candidate variants are not only energetically favorable but also biologically plausible.

Several limitations of the current work should be acknowledged. The GA was originally restricted to 100 generations due to computational constraints, which may have limited the full exploration of the mutational space. The additional 1000-generation experiment addresses this limitation, strengthening our conclusions, but further scaling up of both population size and evolutionary depth will be important for future applications. Moreover, the study focused exclusively on spike protein mutations, without considering other viral proteins or host–pathogen interactions. Functional metrics such as Δ*G* and binding affinity, while informative, may not fully capture the complexity of viral behavior in vivo. Despite these limitations, the study provides a proof-of-concept for applying GA to structural modeling of viral proteins. This methodological framework can serve as a foundation for future research integrating additional fitness functions, larger simulation scales, and more diverse mutational scenarios. Crucially, experimental validation of top candidates (e.g., generation 97) through in vitro assays, as well as benchmarking against genomic surveillance data, will be essential to assess predictive potential. Integration with phylogenetic models, deep learning approaches, and epidemiological studies could further enhance the utility of this approach.

In addition, while Generation 97 emerged as the most optimized candidate in our GA framework, we did not compare its mutations with those observed in circulating VOCs nor assess whether they fall within functionally relevant domains (e.g., receptor-binding domain, N-terminal domain, furin cleavage site). This remains an important limitation of the present study, and a priority for future work integrating genomic surveillance data and structural domain mapping.

Overall, this work demonstrates the promise of GA-based structural modeling as a methodological tool for systematically exploring protein mutations. While not intended to forecast specific variants of concern, it provides a solid computational basis that, once combined with evolutionary and experimental evidence, may contribute to more comprehensive strategies for monitoring viral evolution and informing vaccine design.

## 5 Conclusion

As the COVID-19 pandemic continues and novel variants emerge, computational methods remain essential for understanding the molecular mechanisms that drive viral evolution. Genetic Algorithms (GAs), inspired by natural selection, provide a versatile framework to explore mutational landscapes and refine structural models of viral proteins.

In this study, we implemented a GA-based approach to optimize SARS-CoV-2 spike protein variants using energy-based metrics such as the DOPE score, Gibbs free energy (Δ*G*), and binding affinity. The initial 100-generation run highlighted progressively more stable conformations, with generation 97 representing the most optimized candidate within the explored search space. Extending the GA to 1000 generations confirmed the robustness of this framework, as DOPE scores continued to decrease and revealed long-term convergence patterns, including transient plateaus followed by renewed optimization. These findings illustrate the ability of GAs to iteratively refine structural plausibility and energetic favorability over both short and extended evolutionary timescales.

While the present work does not aim to predict specific real-world variants of concern, it demonstrates the potential of GA-driven modeling as a methodological tool to investigate mutational effects at the structural level. Importantly, the approach establishes a computational foundation that could, in the future, be combined with genomic surveillance, phylogenetics, and experimental validation to strengthen predictive insights.

Future research should build on this framework by expanding fitness functions, adopting multi-objective optimization strategies, and scaling up both the number of generations and the population sizes. Such developments may enhance our ability to link structural modeling with evolutionary dynamics, ultimately supporting more comprehensive strategies for monitoring viral evolution and informing vaccine design.

## Data Availability

Datasets and software used in the experiments are listed as follows: (1) NCBI: National Center for Biotechnology Information (National Center for Biotechnology Information (nih.gov)), (2) RCSB Protein Data Bank: RCSB PDB: Homepage, (3) MODELLER: About MODELLER (salilab.org). All the Python and R scripts used for the analysis are available upon request to the authors.
